# Dynamic hip screws versus cephalocondylic intramedullary nails for unstable extracapsular hip fractures in 2021: A systematic review and meta-analysis of randomised trials

**DOI:** 10.1016/j.jor.2022.12.015

**Published:** 2023-01-08

**Authors:** Siddarth Raj, Sarika Grover, Harroop Bola, Akhilesh Pradhan, Muhammad Ali Fazal, Akash Patel

**Affiliations:** a GKT School of Medical Education, King's College London, London, UK; b University Hospital Coventry and Warwickshire, NHS Trust, Coventry, UK; c Imperial College School of Medicine, Imperial College London, London, UK; d Royal London Hospital, Barts NHS Trust, London, UK; e Department of Trauma and Orthopaedics, Royal Free London NHS Foundation Trust, London, UK; f School of Medicine, University College London (UCL), London, UK

**Keywords:** Dynamic hip screw (DHS), Intramedullary nail (IMN), Extracapsular fracture, Meta-analysis, Systematic review

## Abstract

**Background:**

Extracapsular hip fractures comprise approximately half of all hip fractures and the incidence of hip fractures is exponentially increasing. Extramedullary fixation using a dynamic hip screw (DHS) has been the gold standard method of operative treatment for unstable extracapsular fractures, however, in recent years, intramedullary nails (IMN) have become a popular alternative. IMN versus DHS is continuously discussed and debated in literature. Therefore, the purpose of this systematic review and meta-analysis is to directly compare the peri- and post-operative outcomes of these two techniques to provide an up-to-date analysis of which method of fixation is superior.

**Methods:**

The MEDLINE/PubMed, Embase and Web of Science Database were searched for eligible studies from 2008 to April 2022 that compared peri- and post-operational outcomes for patients undergoing IMN or DHS operations for fixation of unstable extracapsular hip fractures (PROSPERO registration ID:CRD42021228335). Primary outcomes included mortality rate and re-operation rate. Secondary outcomes included operation time, blood loss, transfusion requirement, complication, and failure of fixation rate. The risk of bias and quality of evidence were assessed using the Cochrane RoB 2.0 tool and GRADE analysis tool, respectively.

**Results:**

Of the 6776 records identified, 22 studies involving 3151 patients were included in the final review. Our meta-analysis showed no significant differences between mortality rates (10 studies, OR 0.98; 95% CI 0.80 to 1.22, p = 0.88) or re-operation rates (10 studies, OR 1.03; 95% CI 0.64 to 1.64, p = 0.91) between the two procedures. There were also no significant differences found between complication rates (17 studies, OR 1.29; 95% CI 0.79 to 2.12, p = 0.31) and failure of fixation rates (14 studies, OR 1.32; 95% CI 0.74 to 2.38, p = 0.35). However, DHS operations had a significantly longer operation time (p < 0.0001) and blood loss (p < 0.00001) than IMN operations.

**Conclusion:**

Overall, based on the outcomes assessed, this review has demonstrated that there is no significant difference in the post-operative outcomes for DHS vs IMN, however a significant difference exists in two of the intraoperative outcomes assessed in this review.

## 1 Introduction

Hip fractures are one of the most common injuries affecting patients >65 years and are associated with significant morbidity and mortality. The incidence of hip fractures increases with age and commonly present as fragility fractures that result from osteoporosis.

Due to an increasingly ageing population, the incidence of hip fractures is exponentially expanding and majorly impacting healthcare systems and patients. In 2019, 76,000 patients presented to a hospital in the UK with a hip fracture, an increase from the approximate 65,000 patients that presented in 2017.^1^^,^^2^ Hip fractures also account for 1.8 million hospital bed days per year and cost the NHS £1.1 billion in hospital costs annually, excluding the costs of social care.^3^ Similar trends are seen globally as ageing populations are affecting many countries and by 2050, it is estimated that the annual worldwide incidence of hip fractures will be 6 million.^4^, ^5^, ^6^

Extracapsular hip fractures comprise approximately half of all hip fractures and are usually the result of low-energy mechanisms in elderly patients.^7^^,^^8^ The AO/OTA classification can be used to classify extracapsular hip fractures depending on the relationship of the fracture to the greater and lesser trochanters. Extracapsular hip fractures are classified by AO as Type 31-A and subdivided into groups A1, A2 and A3. Type A1 is a stable trochanteric fracture, type A2 is an unstable trochanteric fracture and type A3 is an unstable transtrochanteric fracture, which includes fractures at the level of the lesser trochanter and reverse oblique patterns.^9^

Extracapsular fractures are generally treated by surgical intervention. Most of the bone in this area is cancellous and highly vascularised in comparison to intracapsular hip fractures, resulting in a robust healing environment suitable for operations.^4^^,^^7^ For the past 40 years, the dynamic (sliding) hip screw (DHS) has been the gold standard method of operative treatment for extracapsular hip fractures.^10^ DHS consists of a lag screw passed into the femoral head which is then attached to a plate, to be secured on the side of the femur, allowing the femoral head component to move along one plane whilst enabling compression at the fracture site.^11^ In the last 20 years, intramedullary nails (IMNs) have become a popular method of fixation as an alternative to DHS, especially for those with unstable fracture patterns. A cephalocondylic IMN is inserted through the greater trochanter or piriform fossa of the femur and is secured by a screw that is passed up from the femoral neck into the femoral head.^12^ They may be biomechanically advantageous for unstable fractures by providing better load sharing.^10^

Intramedullary versus extramedullary fixation is still frequently and controversially discussed and debated in the literature. Older studies (1991–1999) demonstrated that the DHS appeared to be a superior implant to IMN due to lower complication rates and risk of femoral fracture, however, newer studies (2000–2005), utilising newer generations of IMN, demonstrated that IMN did not increase the risk of periprosthetic femoral fracture.^13^ The current NICE guidelines recommend DHS as the surgical treatment for A1 and A2 fractures and IMN for A3 fractures whereas the American Academy of Orthopaedic Surgeons (AAOS) guidelines recommend either DHS or IMN for stable fractures and IMN for unstable fractures.^14^^,^^15^ These guidelines however are still not supported by clinical studies as many recent meta-analyses have demonstrated no notable difference or advantage to choosing DHS in comparison to IMN.^16^, ^17^, ^18^

The purpose of this systematic review is to evaluate more recent randomised controlled trials comparing IMN and DHS in adult patients for the stabilisation of extracapsular hip fractures from 2008 to 2022 to provide a more focused analysis of the outcomes using newer generations of IMN and DHS implants. This review will assess and evaluate the recent evidence for treating adult patients with unstable extracapsular hip fractures using either IMN or DHS to assess which procedure results in better peri- and post-operative outcomes for the patient.

## 2 Methods

The article search and selection for this review were carried out based on the standardised methodology recommended by the Cochrane Methods group for the systematic review of interventions and the Preferred Reporting Items for Systematic Reviews and Meta-Analyses (PRISMA) criteria.

### 2.1 Search strategy

The protocol for this review was prospectively registered on PROSPERO (registration ID CRD42021228335). MEDLINE/PubMed, Embase and Web of Science were searched for eligible studies. The search was limited to studies from 2008 to April 2022. Details of the search strategy have been provided (Appendix A). Two reviewers (SG and SR) performed the search and evaluated titles, abstracts then full-text articles to decide on eligible studies to include. The reference lists of the articles included were also searched for further eligible studies. The Cochrane Risk of Bias 2.0 Tool was used to guide the assessment of the studies identified from the literature search.^19^ For all eligible articles, SG and SR performed data extraction including demographics of participants, study characteristics, procedures and outcomes. Any disagreement was resolved via discussion and any dispute was settled by a consensus involving all authors. The data from eligible articles were inputted into a pre-defined spreadsheet that was reviewed by an additional author (APa).

### 2.2 Eligible studies

Only randomised/quasi-randomised studies comparing peri-operational and post-operational outcomes for patients undergoing operations with cephalocondylic IMN in comparison with DHS for fixation of unstable extracapsular trochanteric hip fractures were included in this review. Duplicate studies, case reports, editorials, letters, and conference proceedings were excluded as per the pre-determined inclusion and exclusion criteria (Table 1).

**Table 1:** Inclusion and exclusion criteria.

Inclusion criteria	Exclusion criteria
● Randomised/quasi-randomised studies ● Skeletally mature patients ● Extracapsular proximal femur fracture ● Intramedullary cephalocondylic nails versus dynamic hip screws ● English language articles only ● Human studies ● Patient outcomes data clearly discussed (mortality, function, complications, reoperation) ● Trials published from 2008 to April 2022 (inclusive)	● Duplicate studies excluded ● Case reports, editorials, comments, letters, guidelines, protocols, abstracts, review papers, demographic studies, unpublished studies ● Anatomical/cadaveric/biomechanical studies ● Trials assessing only pathological or subtrochanteric fractures ● Trials assessing more than 2 methods of fixation

### 2.3 Eligible participants

This systematic review included male or female skeletally mature patients with unstable extracapsular (intertrochanteric or subtrochanteric) hip fractures undergoing treatment with either cephalocondylic IMN or DHS for fixation in the primary setting and therefore excluded those who were undergoing revision surgery.

### 2.4 Eligible interventions and comparators

The eligible intervention included fixation by cephalocondylic IMN of any material and type for fixation of unstable extracapsular hip fractures. The comparator was the use of DHS for fixation of unstable extracapsular fractures of any type and material regardless of technique.

### 2.5 Outcome measures

The primary outcome measures were patient mortality and reoperation rates at final follow-up, measured as percentages. The secondary outcomes were failure of fixation rate, complication rate and surgical outcomes, such as mean operating time, blood loss and transfusion requirement.

### 2.6 Assessment of risk of bias

All randomised control trials included in this study were assessed for risk of bias via the Cochrane Risk of Bias 2.0 tool^19^ and the quality of our effect estimate was analysed using the GRADE ranking system.^20^

### 2.7 Data analysis

All quantitative data for patient mortality and re-operation rates at final follow-up that were available have been included and presented in a table demonstrating primary outcomes. All quantitative data for secondary outcomes including operating time, blood loss, transfusion requirement, complication rate and failure of fixation have been measured as either mean or true values and presented in a table. A quantitative meta-analysis has also been carried out to compare mortality, re-operation, complication, failure of fixation rates, operative time and blood loss between the intervention and comparator using the Review Manager (RevMan 5.4.1) software. A random effects model was used as no fixed effects were assumed. When applicable, mean difference and odds ratios were calculated with confidence intervals provided. Studies that had incomplete data or incomparable outcomes were excluded from the meta-analysis. A full discussion of possible explanations and conclusions from the meta-analysis and tabulated data have been explored in the discussion and conclusion sections.

## 3 Results

**Fig. 1:**
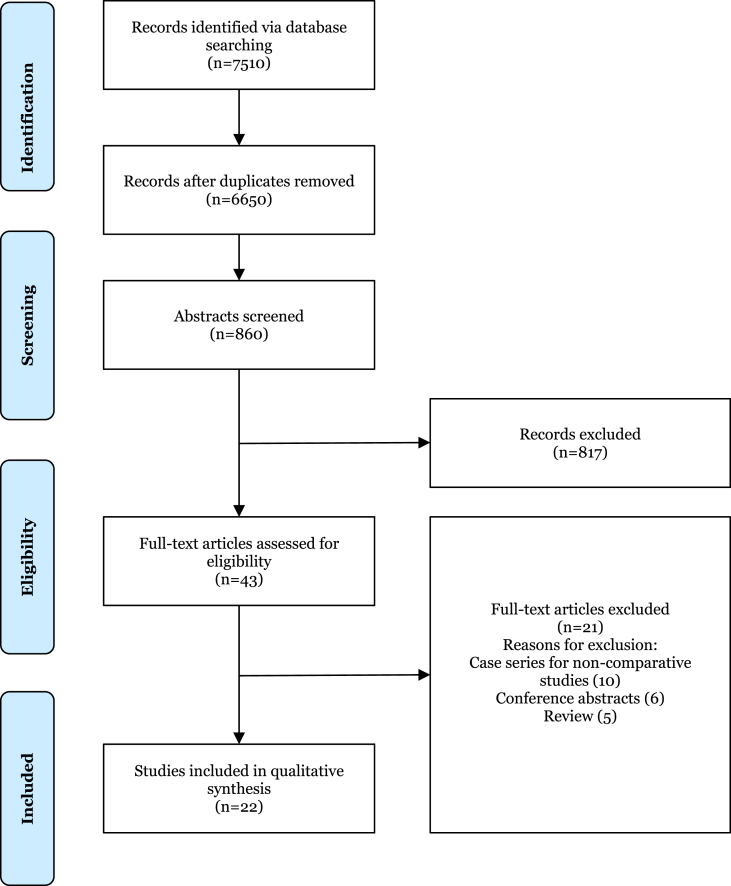
PRISMA flowchart of studies identified, screened, and included.

Following a systematic search, 7510 studies were identified using pre-defined criteria. After the removal of duplicates, 6650 studies remained. 860 abstracts were screened, after which 43 full-text articles were assessed for eligibility, of which 22 studies were included in this review. In accordance with the PRISMA criteria, a flow diagram demonstrating the study selection procedure has been included (Fig. 1). The PRISMA checklist has also been included (Appendix B).

### 3.1 Study characteristics

The baseline characteristics of the included studies are recorded in Table 2. Only studies that compared fixation of unstable extracapsular hip fractures using DHS and IMN were included in this systematic review. Various randomisation techniques were employed across the included studies; in ten studies, randomisation was carried out using sealed envelopes that were computer-generated or generated by a medical statistician.^21^, ^22^, ^23^, ^24^, ^25^, ^26^, ^27^, ^28^, ^29^, ^30^ In three studies, randomisation was carried out by the operating surgeon^31^, ^32^, ^33^ and in another three studies randomisation was carried out using number generators.^34^, ^35^, ^36^ Six studies did not specify a randomisation technique.^37^, ^38^, ^39^, ^40^, ^41^, ^42^ Only one study utilised a single surgeon for all of the operations included in the study.^35^ The patient recruitment period ranged from 2006 to 2019, and all studies were published after 2008. A total of 3151 patients with a mean age of 74.5 (range 58–84) were included; the median number of male and female participants was 25 and 65 respectively. Overall, 1595 and 1556 patients underwent treatment with DHS and IMN, respectively. The types of IMN used included, but was not limited to, gamma nail, intramedullary hip screw and proximal femoral nail. Patients included had 31-A1, 31-A2 or 31-A3 fractures, as classified by the AO/OTA classification. The median follow-up duration was 12 months (range 6–18 months).

**Table 2:** Patient characteristics.

Study	Sample size (number)		Average age (years)		Participants		Fractures Included
	DHS	IMN	DHS	IMN	Male	Female	
**Little et al., 2008**	98	92	84.2	82.6	28	157	31-A1/A2/A3
**Zou et al., 2009**	63	58	65.0	65.0	28	93	31-A1/A2/A3
**Verettas et al., 2009**	60	60	81.0	79.2	35	85	31-A2
**Barton et al., 2009**	110	100	83.3	83.1	44	166	31-A2
**Huang et al., 2010**	48	48	77.0	75.0	25	71	31-A1/A2
**Xu et al., 2009**	55	51	77.9	78.5	31	75	31-A2
**Kumar et al., 2012**	25	25	62.3	62.3	20	30	31-A1/A2/A3
**Matre et al., 2013**	343	341	84.1	84.1	171	513	31-A1/A2/A3
**Nargesh et al., 2013**	48	48	67.0	68.0	26	70	–
**Bhakat et al., 2013**	30	30	67.8	67.8	26	34	31-A2/A3
**Guerra et al., 2014**	19	12	77.9	80.2	6	25	31-A1/A2
**Aktselis et al., 2014**	40	40	–	–	24	56	31-A2
**Chechik et al., 2014**	31	29	83.1	83.1	14	46	31-A1/A2
**Sharma et al., 2015**	15	15	–	–	15	15	31-A2/A3
**Zehir et al., 2015**	102	96	76.9	77.2	76	122	31-A2
**Reindl et al., 2015**	92	112	80.0	82.0	88	116	31-A2
**Neritan et al., 2016**	41	22	77.3	77.3	15	48	31-A1/A2/A3
**Parker et al., 2017**	200	200	83.2	82.0	107	293	31-A1/A2/A3
**Bajpai et al., 2019**	60	60	67.4	66.9	60	60	–
**Eceviz et al., 2020**	27	29	80.8	80.8	26	30	–
**Adeel et al., 2020**	34	34	60.9	59.3	47	21	31-A2/A3
**Saleem et al., 2020**	54	54	60.2	58.5	68	40	–

### 3.2 Primary outcomes

The individual results for the primary outcomes are shown in Table 3. The primary outcomes were mortality rate and reoperation rate for fixation failure at final follow-up. Final-follow up mortality was reported in 11 studies.^21^, ^22^, ^23^, ^24^, ^25^^,^^33^, ^34^, ^35^^,^^37^^,^^40^^,^^41^ None of these studies reported any statistically significant difference in mortality rates at final follow-up between patients that were treated with DHS versus those treated with IMN.

**Table 3:** Primary outcomes (mortality rate, re-operation rate due to failure of fixation).

Study	Final follow-up (months)	Final-follow up mortality			Re-operation rate for fixation failure		
		DHS	IMN	p-value	DHS	IMN	p-value
**Little et al., 2008**	12	17/98 (17.3%)	16/92 (17.4%)	p > 0.05	2/98 (2.0%)	0/92 (0%)	p > 0.05
**Zou et al., 2009**	12	–	–	–	3/63 (4/8%)	0/58 (0%)	–
**Verettas et al., 2009**	–	–	–	–	–	–	–
**Barton et al., 2009**	12	24/110 (21.8%)	32/100 (32.0%)	p < 0.26	2/110 (1.8%)	3/100 (3%)	p < 0.67
**Huang et al., 2010**	9	0/48 (0%)	0/48 (0%)	–	0/48 (0%)	0/48 (0%)	p > 0.05
**Xu et al., 2009**	12	3/55 (5.5%)	2/51 (3.9%)	p > 0.05	1/55 (1.8%)	2/51 (3.9%)	p > 0.05
**Kumar et al., 2012**	12	1/25 (4.0%)	1/25 (4.0%)	p > 0.05	2/25 (8.0%)	0/25 (0%)	p < 0.05
**Matre et al., 2013**	12	87/343 (25.4%)	84/341 (24.6%)	p = 0.83	27/343 (7.9%)	28/341 (8.2%)	p = 0.87
**Nargesh et al., 2013**	12	–	–	–	–	–	–
**Bhakat et al., 2013**	6	–	–	–	2/30 (6.7%)	1/30 (3.3%)	–
**Guerra et al., 2014**	12	8/19 (42.1%)	2/12 (16.7%)	p > 0.05	–	–	–
**Aktselis et al., 2014**	12	5/40 (12.5%)	4/40 (10.0%)	–	–	–	–
**Chechik et al., 2014**	12	1/31 (3.2%)	1/29 (3.4%)	–	1/31 (3.2%)	1/29 (2.4%)	p > 0.05
**Sharma et al., 2015**	6	–	–	–	1/15 (6.7%)	0/15 (0%)	–
**Zehir et al., 2015**	–	26/102 (25.5%)	23/96 (24.0%)	–	0/102 (0%)	0/96 (0%)	p > 0.05
**Reindl et al., 2015**	12	–	–	–	–	–	–
**Neritan et al., 2016**	12	–	–	–	–	–	–
**Parker et al., 2017**	12	59/200 (14.5%)	60/200 (30.0%)	–	0/200 (0%)	3/200 (1.5%)	p = 0.3
**Bajpai et al., 2019**	18	–	–	–	–	–	–
**Eceviz et al., 2020**	12	–	–	–	0/27 (0%)	0/29 (0%)	–
**Adeel et al., 2020**	12	–	–	–	–	–	–
**Saleem et al., 2020**	6	–	–	–	–	–	–

On the other hand, 13 studies reported re-operation rate for fixation failure at final follow-up,^21^, ^22^, ^23^, ^24^, ^25^^,^^28^^,^^31^^,^^33^^,^^35^^,^^37^^,^^38^^,^^40^^,^^42^ of which only one study reported a statistically significant difference (p < 0.05) between DHS and IMN as two out of 25 patients that were treated with DHS required a further operation for fixation failure compared to none out of 25 patients that were treated with IMN.^33^

**Fig. 2:**
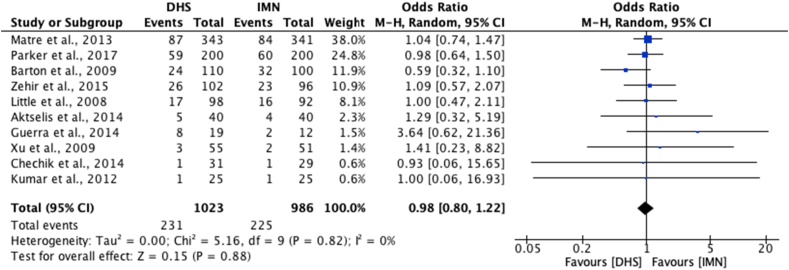
Meta-analysis of mortality rates between DHS and IMN.

**Fig. 3:**
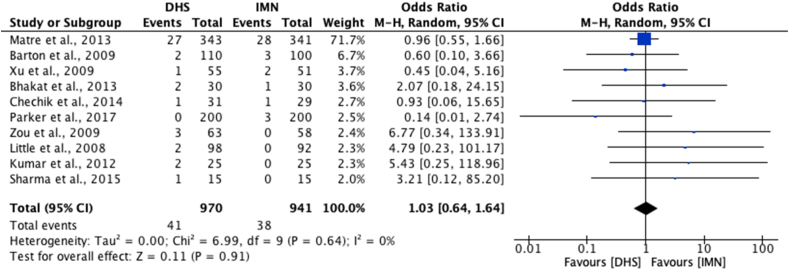
Meta-analysis of re-operation rates between DHS and IMN.

Ten studies^21^, ^22^, ^23^, ^24^, ^25^^,^^33^, ^34^, ^35^^,^^37^^,^^41^ were eligible for meta-analysis of mortality rates at final follow-up (Fig. 2). No significant difference was found between mortality rates for those undergoing DHS in comparison to IMN operations [OR 0.98; 95% CI 0.80 to 1.22, p = 0.88]. On analysis of re-operation rate, 10 studies^21^, ^22^, ^23^, ^24^, ^25^^,^^31^^,^^33^^,^^37^^,^^38^^,^^42^ were eligible for meta-analysis, and similarly, no significant difference was shown between DHS and IMN [OR 1.03; 95% CI 0.64 to 1.64, p = 0.91] (Fig. 3).

### 3.3 Secondary outcomes

The individual results for secondary outcomes are shown in Table 4.

**Table 4:** Secondary outcomes (operation time, blood loss and transfusion requirement, complication rate, failure of fixation rate).

Study	Operation Time (mins)					Mean blood loss (ml)					Mean transfusion requirement (ml)			Complication rate			Failure of fixation rate		
	DHS		IMN		p-value	DHS		IMN		p-value	DHS	IMN	p-value	DHS	IMN	p-value	DHS	IMN	p-value
	Mean	SD	Mean	SD		Mean	SD	Mean	SD										
**Little et al., 2008**	40.3	–	54.0	–	p < 0.001	160.0	–	78.0	–	p < 0.001	–	–	–	19/98 (19.4%)	11/92 (12.0%)	p > 0.05	2/98 (2.0%)	4/92 (4.3%)	p > 0.05
**Zou et al., 2009**	93.0	13.0	52.0	10.0	p < 0.05	410.0	65.0	156.0	24.0	p < 0.05	–	–	–	4/63 (6.3%)	1/58 (1.7%)	p > 0.05	–	–	–
**Verettas et al., 2009**	45.0	–	42.0	–	p = 0.336	200.0	–	150.0	–	p = 0.237	1000	1000	p = 0.847	10/60 (16.7%)	11/60 (18.3%)	–	–	–	–
**Barton et al., 2009**	–		–		–	–		–		–	–	–	–	–	–	–	–	–	–
**Huang et al., 2010**	52.4	18.3	50.5	20.2	–	225.0	–	202.5	–	–	200	200	–	3/48 (6.3%)	5/48 (10.4%)	–	–	–	–
**Xu et al., 2009**	56.5	11.8	68.5	9.9	p < 0.0001	472.9	169.9	220.4	109.9	p < 0.0001	–	–	–	20/55 (36.4%)	13/51 (25.5%)	p > 0.05	1/55 (1.8%)	2/51 (3.9%)	p > 0.05
**Kumar et al., 2012**	87.0	3.2	55.0	18.0	p > 0.05	250.0	44.9	100.0	16.4	p < 0.05	–	–	–	1/25 (4.0%)	1/25 (4.0%)	p > 0.05	2/25 (8.0%)	1/25 (4.0%)	p < 0.05
**Matre et al., 2013**	55.6	–	54.7	–	p < 0.69	263.0	–	180.0	–	p < 0.001	171	143	p = 0.02	21/343 (6.1%)	62/341 (18.2%)	p < 0.001	2/343 (0.6%)	4/341 (1.2%)	p < 0.41
**Nargesh et al., 2013**	65.0	–	42.0	–	–	162.0	–	95.0	–	p < 0.05	–	–	–	6/48 (12.5%)	1/48 (2.1%)	–	1/48 (2.1%)	0/48 (0%)	–
**Bhakat et al., 2013**	69.0	7.3	48.7	2.9	p < 0.0001	213.0	46.4	116.0	19.9	p < 0.0001	–	–	–	2/30 (6.7%)	0/30 (0%)	–	0/30 (0%)	0/30 (0%)	–
**Guerra et al., 2014**	–		–		–	–		–		–	–	–	–	–	–	–	–	–	–
**Aktselis et al., 2014**	75.5	21.9	45.7	22.7	p < 0.001	–		–		–	–	–	–	–	–	–	3/40 (7.5%)	0/40 (0%)	–
**Chechik et al., 2014**	64.0	26.0	54.5	22.5	–	–		–		–	645	360	p = 0.08	9/31 (29.0%)	5/29 (17.2%)	–	6/31 (19.4%)	5/29 (17.2%)	–
**Sharma et al., 2015**	59.7	–	44.5	–	p < 0.05	–		–		–	435	200	p > 0.05	5/15 (33.3%)	1/15 (6.7%)	–	–	–	–
**Zehir et al., 2015**	56.9	5.2	44.4	5.2	p < 0.001	303.1	85.0	139.7	39.7	p < 0.001	–	–	–	64/102 (62.7%)	62/96 (64.6%)	p > 0.05	–	–	–
**Reindl et al., 2015**	–		–		–	–		–		–	–	–	–	–	–	–	2/92 (2.2%)	1/112 (0.9%)	–
**Neritan et al., 2016**	70.8	11.0	49.3	8.8	p < 0.001	122.2	37.2	85.4	25.7	p < 0.001	–	–	–	–	–	–	–	–	–
**Parker et al., 2017**	42.1	11.1	38.3	10.2	p < 0.001	–		–		–	–	–	–	3/200 (1.5%)	2/200 (1.0%)	p > 0.05	2/200 (1.0%)	2/200 (1.0%)	–
**Bajpai et al., 2019**	–		–		–	–		–		–	–	–	–	18/60 (30.0%)	26/60 (43.3%)	–	2/60 (3.3%)	0/60 (0%)	–
**Eceviz et al., 2020**	–		–		–	–		–		–	–	–	–	1/27 (3.7%)	0/29 (0%)	–	0/27 (0%)	0/29 (0%)	–
**Adeel et al., 2020**	58.7	7.8	35.4	5.5	p < 0.05	273.8	30.0	149.8	21.3	p < 0.05	–	–	–	2/34 (5.9%)	1/34 (2.9%)	p > 0.05	6/34 (17.6%)	3/34 (8.8%)	p = 0.283
**Saleem et al., 2020**	78.3	17.1	70.2	16.1	p < 0.013	290.9	73.8	84.7	48.7	p < 0.0000001	–	–	–	7/54 (1.9%)	0/54 (0%)	p < 0.06	3/54 (5.6%)	0/54 (0%)	p < 0.79

#### 3.3.1 Operation time

**Fig. 4:**
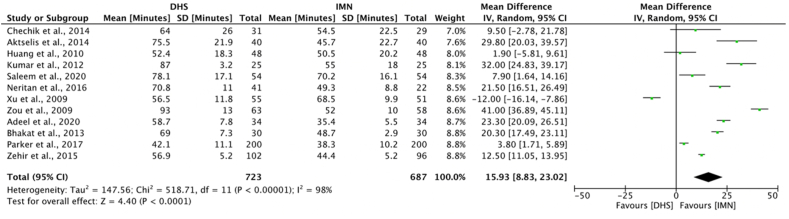
Meta-analysis of operation time between DHS and IMN.

The length of operation was recorded in 17 studies.^22^, ^23^, ^24^, ^25^^,^^27^^,^^29^, ^30^, ^31^, ^32^, ^33^^,^^35^^,^^37^, ^38^, ^39^, ^40^, ^41^, ^42^ One study recorded the greatest difference in operating time between DHS and IMN with an average operation time of 93.0 min for DHS, in comparison to 52.0 min for IMN (p < 0.05).^38^ 12 studies^22^^,^^24^^,^^25^^,^^29^, ^30^, ^31^, ^32^, ^33^^,^^35^^,^^38^^,^^40^^,^^41^ were eligible for meta-analysis (Fig. 4), the results demonstrated that IMN operations were significantly shorter (mean difference 15.93; 95% CI 8.83 to 23.02, p < 0.0001) than DHS operations.

#### 3.3.2 Blood loss and transfusion requirement

**Fig. 5:**
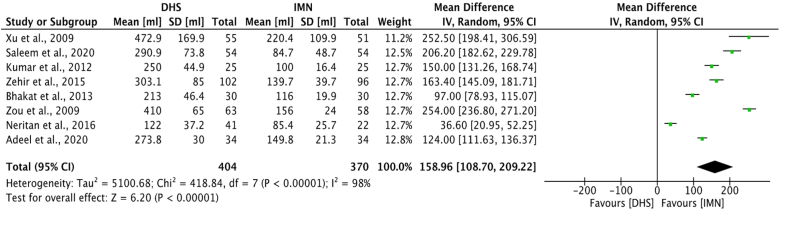
Meta-analysis of blood loss between DHS and IMN.

The mean blood loss during the operation was recorded in millilitres for 13 studies.^22^^,^^23^^,^^27^^,^^29^, ^30^, ^31^, ^32^, ^33^^,^^35^^,^^37^, ^38^, ^39^, ^40^ The mean blood loss ranged from 122.2 ml to 472.9 ml^22^^,^^32^ for DHS and 84.7 ml to 220.4 for IMN.^22^^,^^30^ Eight studies^22^^,^^29^, ^30^, ^31^, ^32^, ^33^^,^^35^^,^^38^ were eligible for meta-analysis (Fig. 5), and it was found that IMN operations led to significantly less intraoperative blood loss (mean difference 158.96; 95% CI 108.70 to 209.22, p < 0.00001) compared to DHS operations. Only 5 studies reported mean transfusion requirement in millilitres^23^^,^^24^^,^^39^^,^^40^^,^^42^ without a consistent timescale for which the transfusion was administered, therefore transfusion requirement was not eligible for meta-analysis.

#### 3.3.3 Complication rate

**Fig. 6:**
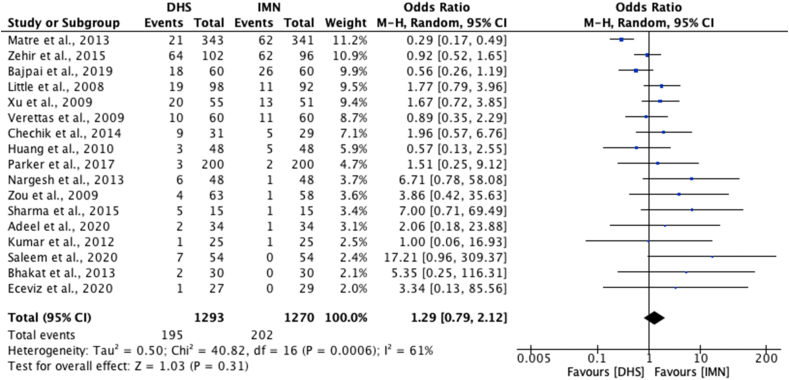
Meta-analysis of complication rates between DHS and IMN.

Complication rates were reported in 17 studies.^22^, ^23^, ^24^, ^25^, ^26^, ^27^, ^28^, ^29^, ^30^, ^31^^,^^33^^,^^35^^,^^37^, ^38^, ^39^, ^40^^,^^42^ Three studies reported a complication rate of 0% for IMN^28^^,^^30^^,^^31^ whereas the lowest reported complication rate for DHS was 1.5%.^25^ The highest reported complication rates were 62.7% for DHS and 64.6% for IMN, both of which were reported in the same study.^35^ Seventeen studies^22^, ^23^, ^24^, ^25^, ^26^, ^27^, ^28^, ^29^, ^30^, ^31^^,^^33^^,^^35^^,^^37^, ^38^, ^39^, ^40^^,^^42^ were included in the meta-analysis of complication rates between DHS and IMN, this revealed no significant difference [OR 1.29; 95% CI 0.79 to 2.12, p = 0.31] (Fig. 6).

#### 3.3.4 Failure of fixation rate

**Fig. 7:**
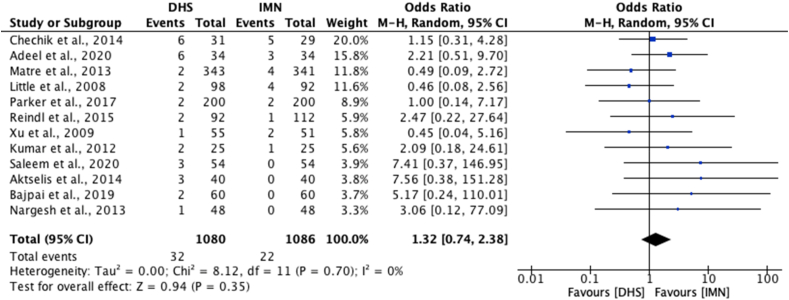
Meta-analysis of failure of fixation rates between DHS and IMN.

Fourteen studies^22^, ^23^, ^24^, ^25^, ^26^, ^27^, ^28^, ^29^, ^30^, ^31^^,^^33^^,^^36^^,^^37^^,^^41^ reported a failure of fixation rate of which only one study^33^ reported a significant difference, where 2 out of 25 DHS patients had a failure of fixation and 1 out of 25 IMN patients had a failure of fixation (p < 0.05). Twelve of these studies^22^, ^23^, ^24^, ^25^, ^26^, ^27^^,^^29^^,^^30^^,^^33^^,^^36^^,^^37^^,^^41^ were eligible for meta-analysis which revealed a non-significant difference in failure of fixation rates between DHS and IMN [OR 1.32; 95% CI 0.74 to 2.38, p = 0.35] (Fig. 7).

### 3.4 Quality assessment

The studies involved in this review were all assessed using the Cochrane Risk of Bias 2.0 Tool and deemed to have some level of bias (Table 5). A GRADE analysis was done for the studies included in the meta-analysis. Failure of fixation revealed a very low overall GRADE rating whereas, re-operation for failure of fixation and complication rate revealed a low rating and mortality rate, a moderate rating (Table 6).

**Table 5:** Risk of bias for randomised comparative studies using the Cochrane RoB 2.0 tool.

Study ID (Author, country and year of publication)	Bias from randomisation	Bias from effect of assignment to intervention	Bias from effect of adhering to intervention	Bias due to missing outcome data	Bias in measurement of outcome	Bias in selection of reported result	Overall risk of bais
**Little et al., 2008**	Some concerns	Low risk	Low risk	Low risk	Low risk	Low risk	Some concerns
**Zou et al., 2009**	Some concerns	Low risk	Low risk	Low risk	Low risk	Low risk	Some concerns
**Verettas et al., 2009**	Some concerns	Low risk	Low risk	High risk	Some concerns	High risk	High risk
**Barton et al., 2009**	Low risk	Low risk	Low risk	Some concerns	Low risk	Some concerns	Some concerns
**Huang et al., 2010**	Some concerns	Low risk	Low risk	Some concerns	Low risk	Some concerns	Some concerns
**Xu et al., 2009**	Low risk	Low risk	Low risk	Low risk	Some concerns	Some concerns	Some concerns
**Kumar et al., 2012**	Low risk	Low risk	Low risk	Some concerns	Some concerns	Some concerns	Some concerns
**Matre et al., 2013**	Low risk	Low risk	Low risk	Low risk	Low risk	Some concerns	Some concerns
**Nargesh et al., 2013**	Low risk	Low risk	Low risk	High risk	Some concerns	High risk	High risk
**Bhakat et al., 2013**	Low risk	Low risk	Low risk	Some concerns	Low risk	Some concerns	Some concerns
**Guerra et al., 2014**	Low risk	Low risk	Low risk	High risk	Some concerns	High risk	High risk
**Aktselis et al., 2014**	High risk	Some concerns	Some concerns	Some concerns	Some concerns	Some concerns	High risk
**Chechik et al., 2014**	Low risk	Low risk	Low risk	Low risk	Low risk	Some concerns	Some concerns
**Sharma et al., 2015**	Some concerns	Low risk	Low risk	Low risk	Low risk	Some concerns	Some concerns
**Zehir et al., 2015**	Low risk	Low risk	Low risk	Some concerns	Some concerns	Low risk	Some concerns
**Reindl et al., 2015**	Low risk	Low risk	Low risk	High risk	Some concerns	Some concerns	High risk
**Neritan et al., 2016**	Some concerns	Low risk	Low risk	Some concerns	High risk	High risk	High risk
**Parker et al., 2017**	Low risk	Low risk	Low risk	Some concerns	Some concerns	Some concerns	Some conerns
**Bajpai et al., 2019**	Low risk	Low risk	Low risk	Some concerns	Some concerns	High risk	High risk
**Eceviz et al., 2020**	Low risk	Low risk	Low risk	Some concerns	Some concerns	Some concerns	Some concerns
**Adeel et al., 2020**	Low risk	Low risk	Low risk	Some concerns	Some concerns	Some concerns	Some concerns
**Saleem et al., 2020**	Low risk	Low risk	Low risk	Some concerns	Some concerns	Some concerns	Some concerns

**Table 6:** Quality of evidence of each outcome using the GRADE analysis.

Outcomes		No. of studies	Risk of bias	Imprecision	Inconsistency	Indirectness	Publication bias	Overall GRADE rating
**Primary**	Mortality rate	10	High	High	High	Moderate	Low	Moderate
	Re-operation for failure of fixation	10	High	Low	Moderate	Low	Low	Low
**Secondary**	Complication rate	17	High	Moderate	Low	Low	Low	Low
	Failure of fixation rate	12	High	Low	Low	Low	Low	Very Low

## 4 Discussion

### 4.1 Summary of findings

This systematic review and meta-analysis has been conducted to provide an up-to-date review to determine which procedure, IMN or DHS, results in better peri-operative and post-operative outcomes. In summary, based on the meta-analysis performed, there was no statistically significant difference in mortality or reoperation rates for either type of operation at final follow-up. There was also no statistically significant difference in complication rate for either procedure as per the meta-analysis. However, the majority of studies included reported that DHS procedures led to significantly higher blood loss and longer operation time than IMN procedures; this was statistically significant in the meta-analysis for these two intra-operative outcomes.

### 4.2 Previous systematic reviews

At the time of writing, this review is the largest systematic review with a meta-analysis that compares mortality and re-operation rates alongside other further adverse outcomes between DHS and IMN procedures. In 2017, a review investigating nail versus plate fixation was published by Parker et al.,^43^ which primarily looked at complications relating to fracture health. Although this review concluded that there was no difference in complication rates for either DHS or IMN procedures, this review only included type A3 fractures and only involved a total of 9 studies. A more recent review published by Lewis et al.^44^ in 2022 compared intramedullary versus extramedullary fixation for extracapsular fractures. Contrary to our review, their primary outcomes were predominantly function-related including: performance of activities of daily living, functional status and health-related quality of life. Although this review involved 76 studies, the review reported that over half of the studies were conducted prior to 2010 and stated that the authors “could not easily judge whether care pathways in these older studies were comparable to current standard of care”.^44^ Moreover, a similar 2022 review also assessed post-operative outcomes including complication rate, non-union, infection or mortality rates between DHS and IMN for AO/OTA subtypes: A1, A2 and A3. The authors investigated each subtype separately and reported difficulty in obtaining data for each one and therefore could not complete a meta-analysis.^18^ Our review therefore adds to the existing literature by providing an up-to-date review that directly compares DHS and IMN procedures for all of A1, A2 and A3 extracapsular fractures collectively and specifically addresses peri-operative as well as post-operative outcomes.

### 4.3 Primary outcomes

In this review, the studies included reported various peri- and post-operative outcomes. Nine studies reported both primary outcomes.^21^, ^22^, ^23^, ^24^, ^25^^,^^33^^,^^35^^,^^40^^,^^43^ Our review found no difference in mortality rate at final follow-up between DHS and IMN procedures. This is in keeping with previous reviews by Wessels et al.^18^ and Zhang et al.,^45^ that also found no difference in mortality rate when comparing DHS to IMN. It has been suggested both procedures could result in a mortality rate of up to 10% in the first year post-procedure, however this could be attributed to the predominantly elderly age group being treated and their existing medical comorbidities.^36^ Our review also demonstrated no significant difference in reoperation rate for fixation failure for either procedure. Alternatively, one study that investigated 17,341 patients demonstrated a lower reoperation rate for IMN at 1 and 3 years in comparison to DHS for unstable femoral fractures.^46^ However, this study investigated reoperations for various other reasons such as implant-related infection, peri-implant fracture, mechanical complications and pain, as opposed to failure of fixation only.

### 4.4 Secondary outcomes

In this review, no single study reported all secondary outcomes and the number of secondary outcomes reported by each study ranged from 0 to 5, meaning that there was marked heterogeneity in the number of secondary outcomes reported. Only a few studies reported the transfusion requirement, therefore it was not possible to carry out a meta-analysis for this outcome. Meta-analysis was completed for the remaining secondary outcomes and although no significant difference was found in complication rate or failure of fixation rate, a significant difference was found in both operation time and blood loss.

The meta-analysis results demonstrated that the operation time was significantly longer for DHS operations and the blood loss was also greater, in comparison to IMN operations. This finding is in keeping with the literature. A previous meta-analysis comparing the same two interventions involving 3097 patients by Hao et al. identified that the operation time was significantly longer and blood loss significantly greater in DHS operations in comparison to IMN.^47^ Another previous meta-analysis recommends the use of IMN for the treatment of unstable intertrochanteric fractures based on the fact that it results in reduced blood loss.^48^ There is speculation to suggest that DHS operations could result in more blood loss and higher infection rate given that they have a longer operative time.^49^

The meta-analysis of the 17 studies that reported complication rate revealed that there was no significant difference in complication rates between either procedure. In keeping with the literature, one study followed approximately 5700 patients over 7 years following DHS or IMN procedures and noted that within 30 days after surgery, the complication rates was exactly 16% for both groups (p = 0.98).^50^ Similarly, a further meta-analysis showed no significant differences in implant-related post-operative complications such as femoral shaft fracture, non-union, breakage of implant and migration of screw between DHS or IMN.^51^ Our review showed no difference in failure of fixation for either procedure. Although failed fixations are rarely reported, a previous study has provided data suggesting that for some unstable fracture patterns including high comminuted fractures or reverse oblique fractures, DHS may be more likely to fail.^52^ Further comparison for specific unstable fracture types would be required before this can be confirmed, as well as investigating complication and failure of fixation over longer follow-up periods.

Even though DHS and IMN procedures provide similar post-operative outcomes such as mortality, complication, and failure of fixation rate, the results from our review have demonstrated that the DHS procedure results in a proportionally greater blood loss and longer operating time in comparison to IMN. This is suggesting that IMN could arguably be a safer treatment option from the intraoperative aspect, however, DHS remains the gold standard operation in the UK. A previous study in the US demonstrated that along with fixation failure rate, implant cost were the most important factors in determining implant choice for unstable intertrochanteric fractures.^53^ Another study conducted in India reported that the cost of an IMN is 7–8 times the cost of DHS and therefore heavily influencing the decision for method of treatment.^54^ Based on this data, in order to justify the increased use of IMN in the UK, a comparison of clinical and cost effectiveness between the two outcomes may be required.

### 4.5 Strengths and limitations

The strengths of this review include a prospective registration of the study protocol as well as an up-to-date literature search. This review also includes a meta-analysis to compare the primary and some of the secondary outcomes. However, the limitations of this study should be considered. Firstly, there have been previous studies^43^^,^^44^ that have suggested that variations of IMN could have different success rates in comparison to DHS, however this was not investigated further in our review. Secondly, this review did not investigate the different surgical techniques for DHS and IMN to assess whether that had any impact on the results. Furthermore, this review also included older studies, from 2008-2010,^21^^,^^22^^,^^37^, ^38^, ^39^, ^40^ which perhaps utilised older techniques and older models of nails and screws, thereby potentially affecting the peri- and post-operative outcomes. Finally, when assessing the risk of bias using the Cochrane Risk of Bias tool, seven studies were deemed to have an overall ‘high risk’ of bias of and the remaining studies were deemed to have ‘some concerns.’

## 5 Conclusion

Overall, based on the studies that were included and outcomes that were assessed, this systematic review has found that there is a difference in two intraoperative outcomes: operation time and blood loss in favour of IMN. However, there is no significant difference in the post-operative outcomes between IMN and DHS. More high-quality studies could be conducted to compare the clinical and cost effectiveness between these two techniques. Additionally, studies could also compare the use of these two techniques for different types of unstable fractures.

## Funding statement

This research did not receive any specific grant from funding agencies in the public, commercial, or not-for-profit sectors.

## Ethics approval and consent to participate

Not applicable.

## Consent for publication

Not applicable.

## Availability of data and material

The datasets supporting the conclusions of this review are included within the review and its additional files.

## Authors’ contributions

SR and SG performed the search and evaluated titles, abstracts, and then full-text articles to decide eligible studies to include. For all eligible articles SR and SG performed data extraction including demographics of participants, study characteristics, procedures and outcomes. Disagreement was resolved via discussion and where no agreement was reached, a third independent party acted as an arbiter (APa). SR, SG, HB and APr coordinated and equally contributed to writing the manuscript. MAF and APa oversaw, reviewed, and edited the manuscript. All authors read and approved the final manuscript.

## Author statement

**Siddarth Raj:** Conceptualisation, Methodology, Formal Analysis, Investigation, Writing - Original Draft, Writing - Review & Editing. **Sarika Grover**: Conceptualisation, Methodology, Formal Analysis, Investigation, Writing - Original Draft, Writing - Review & Editing. **Harroop Bola:** Writing - Original Draft, Writing - Review & Editing. **Akhilesh Pradhan**: Validation, Writing - Review and Editing, Visualisation. **Akash Patel**: Validation, Visualisation, Supervision, Project Administration, Writing - Review and Editing. **Muhammad Ali Fazal**: Supervision, Project Administration.

## Declaration of competing interest

All authors declare that they have no competing interests.
