# Supplementary material for: Dynamic hip screws versus cephalocondylic intramedullary nails for unstable extracapsular hip fractures in 2021: A systematic review and meta-analysis of randomised trials
Source: J Orthop. 2023 Jan 8;36:88–98. doi: 10.1016/j.jor.2022.12.015 (PMC9841034; doi:10.1016/j.jor.2022.12.015)
Supplement: Multimedia component 1 [file mmc1.docx]

**Appendix A**

Databases and criteria

The MEDLINE/Pubmed, Embase and Web of Science Database will be searched for eligible studies in April 2022. The search will be limited to studies published from the year 2008 onwards to reflect modern practice including the use of newer generations of intramedullary nails. The search will be carried out following the Preferred Reporting Items for Systematic Reviews and Meta-analyses (PRISMA) criteria.

Example of search strategy

1 hip or hip joint

2 proximal femur or proximal femoral

3 intertrochanteric

4 extracapsular

5 1 or 2 or 3 or 4

6 fracture

7 hip fracture

8 6 or 7

9 5 and 8

10 dynamic hip screw or DHS

11 sliding hip screw

12 screw

13 intramedullary nail or IMN

14 cephalocondylic nail

15 proximal femoral nail

16 proximal femoral nail antirotation

17 nail

18 10-17

19 (9 and 18).ti, ab, kw.

[(hip or hip joint OR proximal femur or proximal femoral OR intertrochanteric OR extracapsular) AND (fracture OR hip fracture) AND (dynamic hip screw or DHS OR sliding hip screw OR screw OR intramedullary nail or IMN OR cephalocondylic nail OR proximal femoral nail OR proximal femoral nail antirotation OR nail)]
